# Quantitative Analysis of UV-B Radiation Interception and Bioactive Compound Contents in Kale by Leaf Position According to Growth Progress

**DOI:** 10.3389/fpls.2021.667456

**Published:** 2021-07-08

**Authors:** Hyo In Yoon, Hyun Young Kim, Jaewoo Kim, Jung Eek Son

**Affiliations:** ^1^Department of Agriculture, Forestry and Bioresources (Horticultural Science and Biotechnology), Seoul National University, Seoul, South Korea; ^2^Research Institute of Agriculture and Life Sciences, Seoul National University, Seoul, South Korea

**Keywords:** flavonoids, phenolic content, plant structure, three-dimensional analysis, ultraviolet yield, vertical farm

## Abstract

UV-B (280–315 nm) radiation has been used as an effective tool to improve bioactive compound contents in controlled environments, such as plant factories. However, plant structure changes with growth progress induce different positional distributions of UV-B radiation interception, which cause difficulty in accurately evaluating the effects of UV-B on biosynthesis of bioactive compounds. The objective of this study was to quantitatively analyze the positional distributions of UV-B radiation interception and bioactive compound contents of kales (*Brassica oleracea* L. var. *acephala*) with growth progress and their relationships. Short-term moderate UV-B levels did not affect the plant growth and photosynthetic parameters. Spatial UV-B radiation interception was analyzed quantitatively by using 3D-scanned plant models and ray-tracing simulations. As growth progressed, the differences in absorbed UV-B energy between leaf positions were more pronounced. The concentrations of total phenolic compound (TPC) and total flavonoid compound (TFC) were higher with more cumulative absorbed UV-B energy. The cumulative UV energy yields for TFC were highest for the upper leaves of the older plants, while those for TPC were highest in the middle leaves of the younger plants. Despite the same UV-B levels, the UV-B radiation interception and UV-B susceptibility in the plants varied with leaf position and growth stage, which induced the different biosynthesis of TFC and TPC. This attempt to quantify the relationship between UV-B radiation interception and bioactive compound contents will contribute to the estimation and production of bioactive compounds in plant factories.

## Introduction

*Brassica* vegetable crops are known to have beneficial effects on human health (Heimler et al., 2007; Podsędek, 2007; Francisco et al., 2017). Among them, kale (*Brassica oleracea* L. var. *acephala*), which is a rich source of health-promoting phytochemicals, such as polyphenols and carotenoids (Olsen et al., 2009; Walsh et al., 2015), has been widely cultivated and consumed for several centuries (Šamec et al., 2019). Recently, to improve individual phytochemicals, the nutritional contents and profiles of *Brassica* vegetables have been studied (Krumbein et al., 2010; Ishida et al., 2014). As strategies for optimizing and balancing these profiles, manipulation of environmental factors has been attempted (Oh et al., 2009; Davies and Espley, 2013). Variations in the amounts and patterns of compounds have been attributed to abiotic stress factors, including temperature, drought, salinity, and ultraviolet (UV) radiation (Ramakrishna and Ravishankar, 2011; Linić et al., 2019; Podda et al., 2019; Toscano et al., 2019). In particular, UV-B (280–315 nm) has a great impact on plant defense mechanisms and is used as an effective tool to increase bioactive compound contents over short-term periods in various crops (Czégény et al., 2016; Escobar-Bravo et al., 2017; Yavaş et al., 2020).

Many previous studies have focused on the effects of UV-B energy (dosage) levels on bioactive compounds in many crops for application in controlled environments, such as plant factories (Martínez-lüscher et al., 2013; Hectors et al., 2014; Zhao et al., 2020). UV-B levels have been determined from the energy emitted by UV-B light sources (Bian et al., 2014; Acharya et al., 2016). However, plant responses to UV-B are driven not by the energy released but by the energy absorbed by leaves (Meyer et al., 2009), which changes with leaf position (Morales et al., 2011). UV-B exposure on upper leaves was more advantageous for absorbing light, but lower leaves had no choice but to receive the transmitted light in the plant canopy (Filella and Peñuelas, 1999). In *Arabidopsis*, the light capture efficiency of simulated leaves with spiral phyllotaxis increased with leaf order (Strauss et al., 2020). Therefore, the accumulation pattern of UV-B absorbing compounds should be considered with respect to leaf position (Grammatikopoulos et al., 1999; Liakoura et al., 2003).

In most previous studies, accumulation of bioactive compounds according to leaf position has been interpreted as an influence of UV-B susceptibility that is related to leaf age (Behn et al., 2011; Majer and Hideg, 2012; Holub et al., 2019). Considering the three-dimensional (3D) plant structure, bioactive compounds at each leaf were determined with UV-B radiation interception as well as with leaf age in kale (Yoon et al., 2021). As plant growth progresses, changes in lighting distance (Kim et al., 2020b) or planting density (Portes and Melo, 2014; Xue et al., 2015) can affect the spatial distribution of light interception for whole plants, which is directly related to plant growth and biomass production. Accumulation of bioactive compounds is regulated by the overall developmental stages of whole plants as well as by the specific developmental state of each leaf (Lois, 1994). In particular, under UV-B exposure, secondary metabolites are affected by plant developmental ages in pak choi (Heinze et al., 2018). Therefore, plant growth progress may cause large variations in absorbed UV-B based on leaf position as well as the UV sensitivity due to leaf age. Their influences on metabolite accumulation cannot be distinguished without quantification of the absorbed UV-B energy distribution.

Recently, the light distribution for a whole plant was quantitatively analyzed using 3D plant models and ray-tracing simulation analysis (Kim et al., 2020a,b). However, spatial analysis of light distribution has not been applied to UV-B radiation. These methods allow interpretations of absorbed UV-B distributions based on plant structure. This study hypothesized that, for individual plants, light interception depends on leaf position as well as growth stage, which will affect UV-B-induced biosynthesis of bioactive compounds. The objective of this study was to quantitatively analyze the positional distributions of UV-B radiation interception and bioactive compound contents of kales with growth progress and their relationships using 3D analysis.

## Materials and Methods

### Plant Materials and Growth Conditions

**Figure 1:**
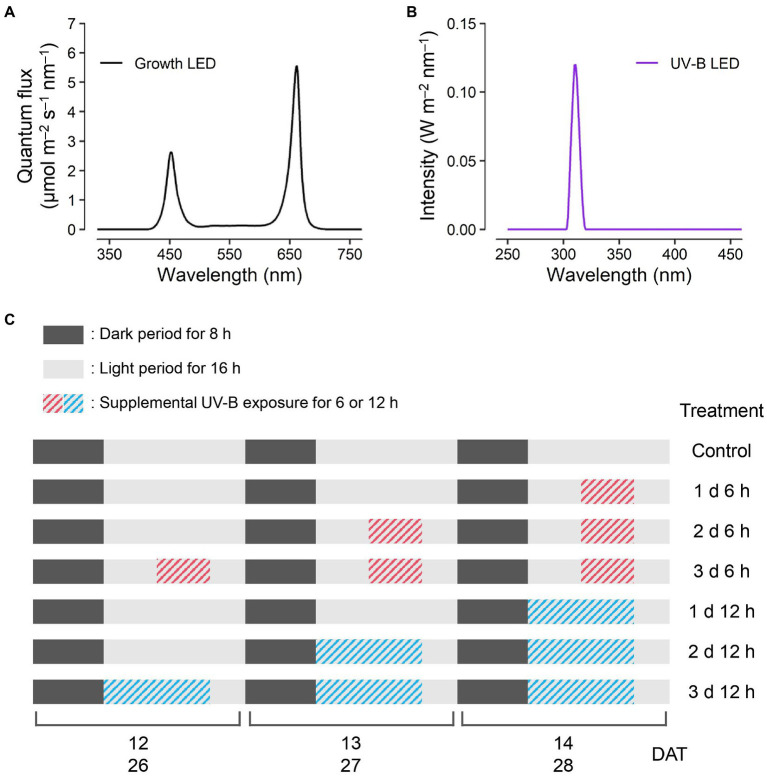
Experimental design. Light spectra of the red, blue, and white light-emitting diodes (LEDs) for plant growth **(A)** at 200 μmol m^−2^ s^−1^ over a waveband of 400–700 nm and UV-B LED **(B)** of 1.2 W m^−2^ at a spectrum peak of 310 nm. The schedules of UV-B treatments **(C)** consisting of supplemental UV-B exposures of 6 and 12 h per day for 1, 2, and 3 days before harvest were the same for both harvest dates at 14 and 28 days after transplanting (DATs).

Kale seeds (*B. oleracea* L. var. *acephala*, “Manchoo collard,” Asia Seed Company, Seoul, South Korea) were sown and germinated on sponge cubes by hydroponic methods under fluorescent lamps at a photosynthetic photon flux density of 150 μmol m^−2^ s^−1^. After the first leaf appeared, the seedlings were supplied with a nutrient solution for *Brassica* modified from a previous study (Choi et al., 2005): N 137.8, P 30.9, K 140.9, Ca 104.6, Mg 54.8, Fe 2.76, Cu 0.02, Zn 0.05, Mn 0.68, B 0.50, and Mo 0.01 mg l^−1^, at an electrical conductivity (EC) of 0.6 dS m^−1^. At 4 weeks after germination, seedlings were transplanted into plant factory modules with a deep flow technique system, and each module was 150 H × 80 W × 50 L (cm) in size. The modules were maintained at 24°C/20°C light/dark temperatures, 70% relative humidity, and 500 μmol mol^−1^ CO_2_ concentration. The transplanted plants were irradiated with light-emitting diodes (LEDs) at 200 μmol m^−2^ s^−1^ over a waveband of 400–700 nm for a 16 h light period and were supplied with 1.2 dS m^−1^ EC nutrient solution. The spectrum of the LED for growth was measured using a spectroradiometer (Blue-Wave spectrometer, StellarNet Inc., Tampa, FL, United States) in the range of 380–900 nm (Figure 1A). Three plants per treatment were harvested at 14 days after transplanting (DAT), and two plants per treatment were harvested at 28 DAT.

### Growth Characteristics

Fresh leaf weights were determined at harvest with three replicates per treatment, and dry leaf weights were measured after drying in an oven for 120 or 168 h for the 14 DAT and 28 DAT plants, respectively. After photographing all leaves, total plant leaf areas were calculated with the image analysis software ImageJ (National Institutes of Health, Bethesda, MD, United States). Leaf positions (upper, middle, and lower leaves) were determined in the order from youngest to oldest, and consisted of 3, 2, and 2 leaves at 14 DAT and 3–4, 3, and 3 leaves at 28 DAT, respectively.

### UV-B Treatment

All plants were irradiated with supplemental UV-B LEDs (Ericson Company Ltd., Bucheon, South Korea) with 1.2 W m^−2^ at a spectrum peak approximately 310 nm. The irradiance and spectrum of UV-B LEDs were measured by a UV sensor (MU-200, Apogee Instruments Inc., Logan, UT, United States) and the spectroradiometer in the range of 250–400 nm (Figure 1B). UV-B exposure periods were 6 and 12 h per day for 1, 2, and 3 days before harvest, which resulted in a total of six treatments (e.g., 1 d 6 h, 1 d 12 h, 2 d 6 h, 2 d 12 h, 3 d 6 h, and 3 d 12 h), and the experimental setup is shown in Figure 1C. The cumulative UV-B doses of six treatments were 21.6, 43.2, 43.2, 86.4, 64.8, and 129.6 kJ m^−2^ for 3 days, respectively, which are equivalent to biologically effective UV-B radiation (UV-B_BE_) doses of 2.1, 4.2, 4.2, 8.4, 6.3, and 12.6 kJ m^−2^ for 3 days, respectively. UV-B_BE_ was calculated using a plant action spectrum in the UV range (Flint and Caldwell, 2003). After UV-B exposure, all plants had a recovery time of 4 h per day. At the end of 14 and 28 DAT, the plants from all treatments were harvested simultaneously.

### Chlorophyll Fluorescence

Chlorophyll fluorescence was measured for 30 min dark-adapted leaves using a chlorophyll fluorescence meter (Handy PEA fluorometer, Hansatech, Kings Lynn, United Kingdom) according to a previous study (Rapacz, 2007). At 14 and 28 DATs, all measurements were performed after the recovery time with three replicates per treatment and leaf position. Measurements were performed using a saturating pulse of 1,500 μmol m^−2^ s^−1^ for 1 s to determine the minimal fluorescence (*F*_0_) and maximal fluorescence (*F_m_*) values. The maximal photochemical efficiency of photosystem II (*F_v_*/*F_m_*) was calculated as (*F_m_ − F*_0_)/*F_m_*.

### Bioactive Compounds and Antioxidant Capacity

#### Total Phenolics

Total phenolic compounds (TPCs) were measured by the Folin-Ciocalteu colorimetric method (Ainsworth and Gillespie, 2007). Powdered samples (50 mg) were mixed with 1 ml of 80% methanol, incubated for 48 h in the dark at room temperature and centrifuged at 1.1 × 10^4^ *g* for 10 min. Supernatants of samples (50 μl) were collected in 2 ml microtubes and were then mixed with 750 μl of 10% Folin-Ciocalteu solution and 135 μl of distilled water using a vortexer. After mixing, 600 μl of 700 mm Na_2_CO_3_ was added, and the samples were then incubated for 2 h at room temperature. Sample absorbances were read at 765 nm using a spectrophotometer (PhotoLab 6100 VIS, Weilheim, Germany). The standard unit for TPC was expressed as milligrams of gallic acid equivalent per gram of dry weight (mg GAE g^−1^ DW).

#### Total Flavonoids

Total flavonoid compound (TFC) amounts were measured by an aluminum chloride colorimetric method (Dewanto et al., 2002; Lee et al., 2012). Powdered samples (50 mg) were mixed with 1 ml of 80% methanol, incubated for 24 h in the dark at 4°C, and centrifuged at 1.1 × 10^4^ *g* for 10 min. The supernatants (50 μl) of the samples were collected in 2 ml microtubes, and 135 μl of distilled water and 45 μl of 5% NaNO_2_ were added. Ninety microliters of 10% AlCl_3_ and 300 μl of 1 M NaOH were added after 5 and 6 min, respectively, and 165 μl of distilled water was then added. After incubating for 30 min, sample absorbances were then read at 510 nm using a spectrophotometer, and the standard unit for TFC was expressed as milligrams of catechin acid equivalent per gram of dry weight (mg CE g^−1^ DW).

#### Antioxidant Capacity

Total antioxidant capacity was measured using the 2, 2-diphenyl-1-picrylhydrazyl (DPPH) assay method (Brand-Williams et al., 1995; Andarwulan et al., 2010). A DPPH solution was prepared with 500 ml of 80% methanol and 12 mg of DPPH. Powdered samples (50 mg) were mixed with 1 ml of 80% methanol, incubated for 48 h in the dark at room temperature and centrifuged at 1.1 × 10^4^ *g* for 10 min. Supernatants (50 μl) were then collected in 2 ml microtubes, and 1.95 ml of DPPH solution was added. After incubating for 30 min, the sample absorbances were then read at 517 nm by the spectrophotometer and used methanol as the blank. Antioxidant activity was expressed as radical scavenging activity (RSA), which was calculated using the following equation:

(1) $$RSA\;\left(\%\right)=\left(A_{control\;517nm}-A_{sample\;517nm}\right)/A_{control\;517nm}\times 100$$

where the *A*_control 517 nm_ and *A*_sample 517 nm_ are the absorbances of the samples at 517 nm without and with leaf extracts, respectively.

### Light Interception With 3D Plant Structure

Light interceptions of kale plants were analyzed using 3D-scanned plant models and ray-tracing simulation analysis method (Kim et al., 2020a,b; Yoon et al., 2021). The detailed procedure and condition from scan to simulation were described in Supplementary Figure S1.

#### Construction of 3D-Scanned Plant Models

The plants were scanned with a high-resolution portable 3D scanner (GO!SCAN50TM, CREAFORM, Lévis, Quebec, Canada). The scanner resolution was set at 2 mm. The scanned plants were randomly selected as one plant per treatment (Control, UV_6 h_ and UV_12 h_) before and after treatment, and a total of six scanned models were generated at each growth stage. The scanned data were exported to 3D mesh data with its scanning software (VXelement, CREAFORM). Holes and noise in the mesh data were fixed, and segmented mesh data were reconstructed to a surface model to perform ray-tracing simulations by reverse engineering software (Geomagic Design X, 3D Systems, Rock Hill, SC, United States).

#### Ray-Tracing Simulation

For the ray-tracing simulations, the transmittance and reflectance of each leaf position and module material were measured using the spectroradiometer with an integrating sphere (IC-2, StellarNet Inc.) in a range of 250–700 nm to determine optical properties for the plant models (Supplementary Figure S1). A virtual growth bed and LED bars were reconstructed using 3D computer-aided design software (Solidworks, Dassault Systèmes, Vélizy-Villacoublay, France) with the same size and layout as the actual growth environment. Twenty-four or twelve surface models of scanned plants were placed on the virtual growth bed equal to the actual planting density for each growth stages, 14 or 28 DAT, respectively. Ray-tracing simulations were performed by using a ray-tracing software (Optiworks, OTIS Inc., La Farlède, France). After setting up all leaf surface models as separate detectors, the simulation outputs were averaged according to leaf position and treatment. All simulation results are presented as the average light interceptions in the photosynthetically active radiation (PAR) range of 400–700 nm and the UV range of 250–400 nm.

### Statistical Analysis

Comparison of mean value were performed with one-way or two-way ANOVA and Tukey’s HSD test to assess the effects of treatments or leaf positions with R software (R 1.2.5, R Foundation, Vienna, Austria). The UV energy yields for TPC and TFC contents were considered as the increase rate based on cumulative absorbed UV energy, and the increase rates compared to the control were regressed into a nonlinear regression as follows:

(2) $$Increase\;rate\;of\;TPC\;or\;TFC\;\left(\%\right)=a/\Delta UV+b$$

where *a* and *b* are the regression coefficients for the relationships between bioactive compounds and absorbed UV. ∆*UV* is the UV absorbed on leaves in each treatment. Linear and nonlinear regressions were conducted with Python (Python 3.6.7, Python Software Foundation, Wilmington, United States).

## Results

### Plant Growth

**Figure 2:**
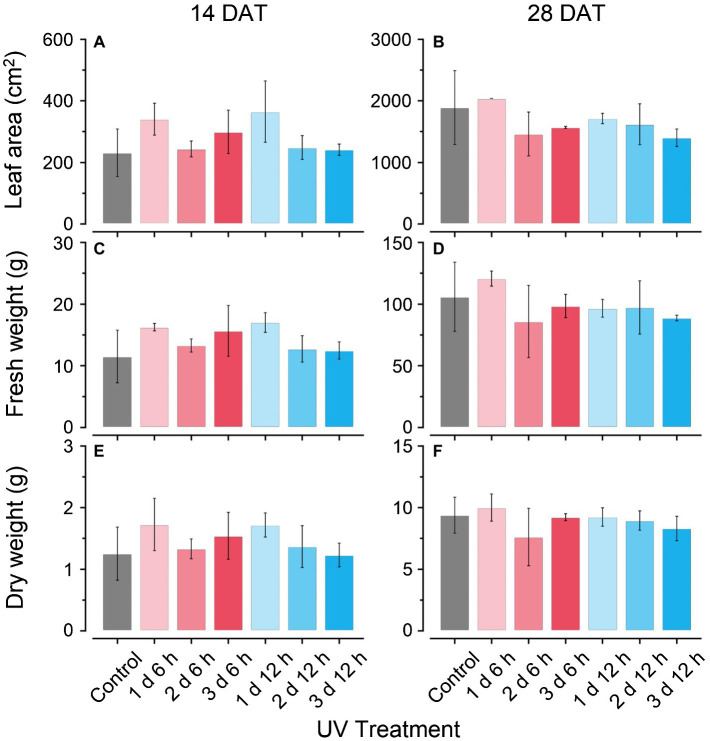
Total leaf areas **(A,B)**; leaf fresh weights **(C,D)**; and dry weights **(E,F)** of kales grown under the control and UV-B treatments at 14 and 28 days after transplanting (DATs). Vertical vars indicate the standard deviation of mean. Refer to Figure 1C for the UV treatments.

The plant growth characteristics did not show any significant differences among the treatments at either growth stage during cultivation (Figure 2). In the UV_12 h_ treatments (e.g., 1 d 12 h, 2 d 12 h, and 3 d 12 h), fresh leaf weights and dry leaf weights slightly decreased with the UV-B exposure period at 28 DAT (Figures 2C–F), and leaf areas were 10–25% lower than those of the control (Figures 2A,B). *F_v_*/*F_m_* values at 14 and 28 DATs were 0.82–83 in all treatments and did not differ among treatments (data not shown). Therefore, UV-B levels did not affect the growth or photochemical efficiency in all treatments.

### Vertical Distributions of PAR and UV Radiation Interception

**Figure 3:**
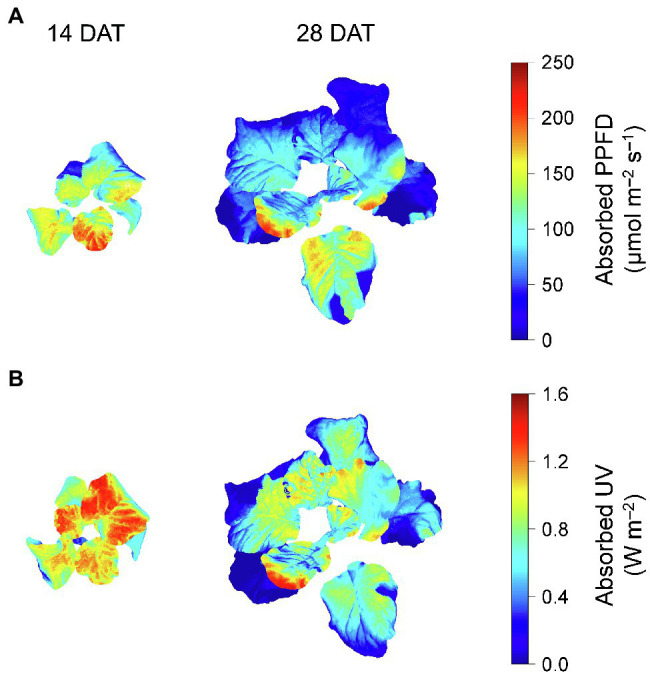
Representative light interception distributions on 3D-scanned kale models at 14 and 28 days after transplanting (DATs). Each light interception represents the photosynthetic photon flux density (PPFD) absorbed at at 400–700 nm **(A)** and UV absorbed at 250–400 nm **(B)**.

**Figure 4:**
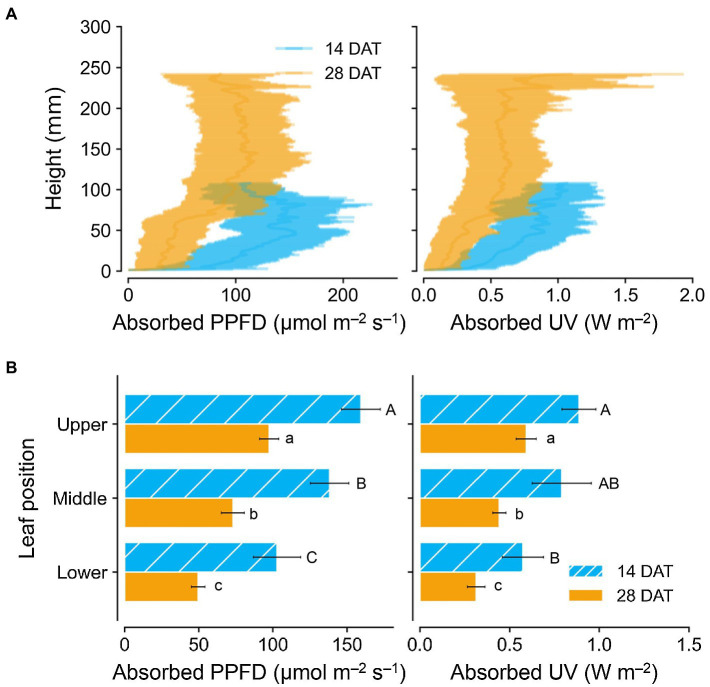
Vertical distributions of light interception on kales according to plant heights **(A)** and leaf positions **(B)** at 14 and 28 days after transplanting (DATs). Each light interception represents the photosynthetic photon flux density (PPFD) absorbed at 400–700 nm and UV absorbed at 250–400 nm. Vertical vars indicate the standard error of mean (*n* = 2; average of 12 and 6 plant models for 14 and 28 DATs, respectively). Different letters represent significant differences between leaf position at *p* < 0.05 by two-way ANOVA and Tukey’s HSD test.

Photosynthetically active radiation and UV radiation interceptions were simulated well with the 3D structure of the plants, i.e., leaf positions and leaf angles (Figure 3). Radiation interception levels increased with plant height and leaf position in the order of upper, middle, and lower leaves for both growth stages (Figure 4). Plants at 14 DAT had lower plant heights but the leaves at each position received higher light intensities compared to those at 28 DAT (Figure 4A). At 14 DAT, the absorbed UV levels of the upper leaves were 12.1 and 54.5% higher than those of the middle and lower leaves, respectively (Figure 4B). The differences in absorbed UV light between leaf positions were more pronounced at 28 DAT, for which the UV radiation interceptions of the upper leaves were 34.1 and 88.8% higher than those of the middle and lower leaves, respectively.

### Bioactive Compounds and Antioxidant Capacity

**Figure 5:**
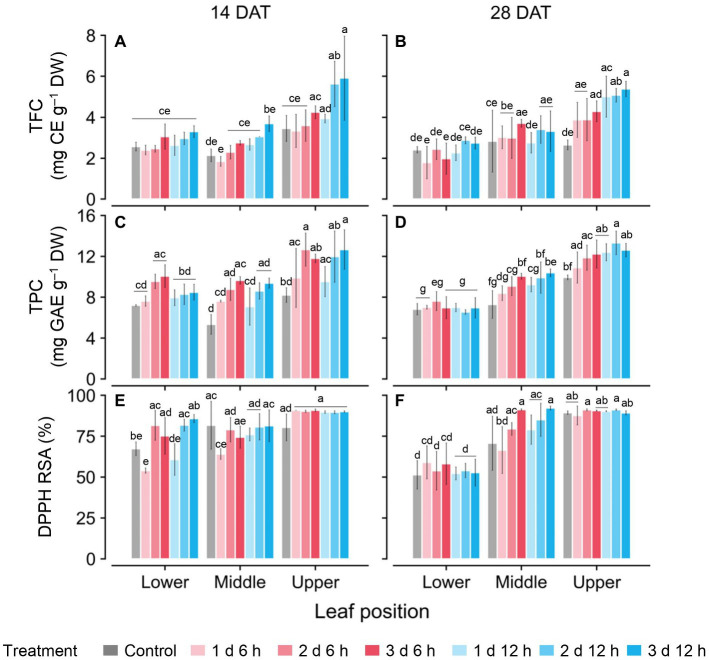
Concentrations of total flavonoid compounds (TFC; **A,B**); total phenolic compounds (TPC; **C,D**); and antioxidant capacity represented as DPPH radical scavenging activities **(E,F)** of kales grown under control and UV-B treatments according to leaf positions at 14 and 28 days after transplanting (DATs). Vertical vars indicate the standard deviation of mean, *n* = 3. Different letters represent significant differences for each parameter at *p* < 0.05 by two-way ANOVA and Tukey’s HSD test. Refer to Figure 1C for the UV-B treatments.

TFC and TPC concentrations and antioxidant capacity (RSA) were higher with greater UV-B doses and higher leaf positions (Figure 5). Only on the upper leaves at 14 DAT were the TFC and TPC levels significantly higher than those at other positions (Figures 5A,C,E), while those at 28 DAT increased significantly in the order of upper, middle, and lower leaves (Figures 5B,D,F). In all treatments at 14 DAT, the TFC and TPC levels in the upper leaves were 35.9–63.1% and 29.6–55.2% higher than those of the other leaves, respectively. At 28 DAT, those values in the upper leaves were 29.3–36.8% and 70.1–82.6% higher than those of the other leaves, respectively. Overall, the TFC, TPC, and RSA values for the 2 d 12 h and 3 d 12 h treatments were higher than those for the control at both growth stages.

### Relationships Between UV Light Interception and Bioactive Compounds

**Figure 6:**
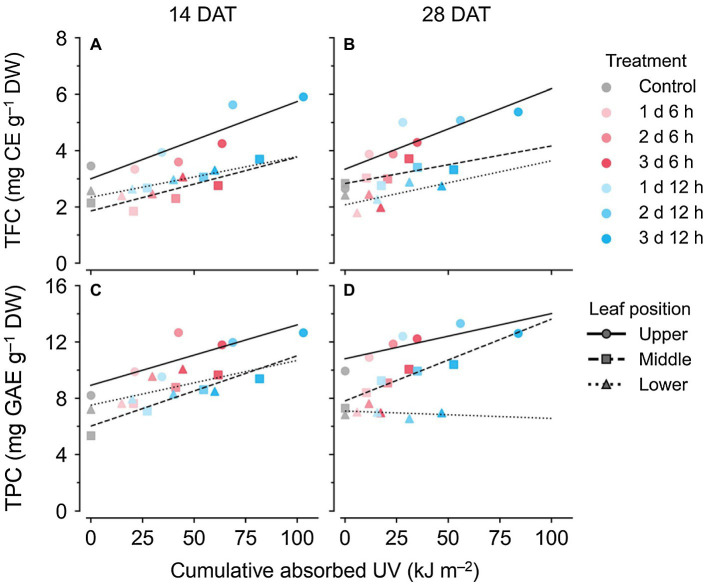
Relationships between compound concentrations and cumulative absorbed UV for 3 days of kales grown under the control and UV-B treatments according to leaf position and growth stage; total flavonoid compounds (TFC; **A,B**) and total phenolic compounds (TPC; **C,D**) at 14 **(A,C)** and 28 **(B,D)** days after transplanting (DATs). The cumulative absorbed UV means the integration of UV irradiation absorbed by each leaf for 3 days. Refer to Figure 1C for the UV-B treatments and Table 1 for R^2^ of the linear regression.

Across all data, the TPC and TFC concentrations relative to the cumulative amounts of UV absorbed for 3 days showed linear relationships at each leaf position (Figure 6). The coefficients of determination (*R*^2^) of the linear regressions are shown in Table 1. As growth progressed from 14 to 28 DATs, the gradients of TFC levels against absorbed UV increased slightly for the upper leaves (Figures 6A,B). In contrast, the gradients of TPC levels for the upper leaves decreased by 25% but only increased by 13.8% in the middle leaves at 28 DAT compared to those at 14 DAT.

**Table 1:** Coefficients of determination (*R*^2^) from linear regressions for bioactive compound contents and from nonlinear regressions for change rates with cumulative absorbed UV for 3 days according to leaf position and growth stage. Refer to Eq. 2 for the nonlinear regression equation.

Growth stage	Leaf position	Linear regression		Nonlinear regression	
		TFC^z^	TPC^y^	TFC	TPC
14 DAT^x^	Upper	0.79	0.70	0.64	0.59
	Middle	0.73	0.84	0.66	0.74
	Lower	0.73	0.39	0.71	0.37
28 DAT	Upper	0.73	0.64	0.59	0.84
	Middle	0.45	0.87	0.34	0.91
	Lower	0.41	0.07	0.61	0.11

z TFC, total flavonoid compounds;

y TPC, total phenolic compounds; and

x DAT, days after transplanting.

**Figure 7:**
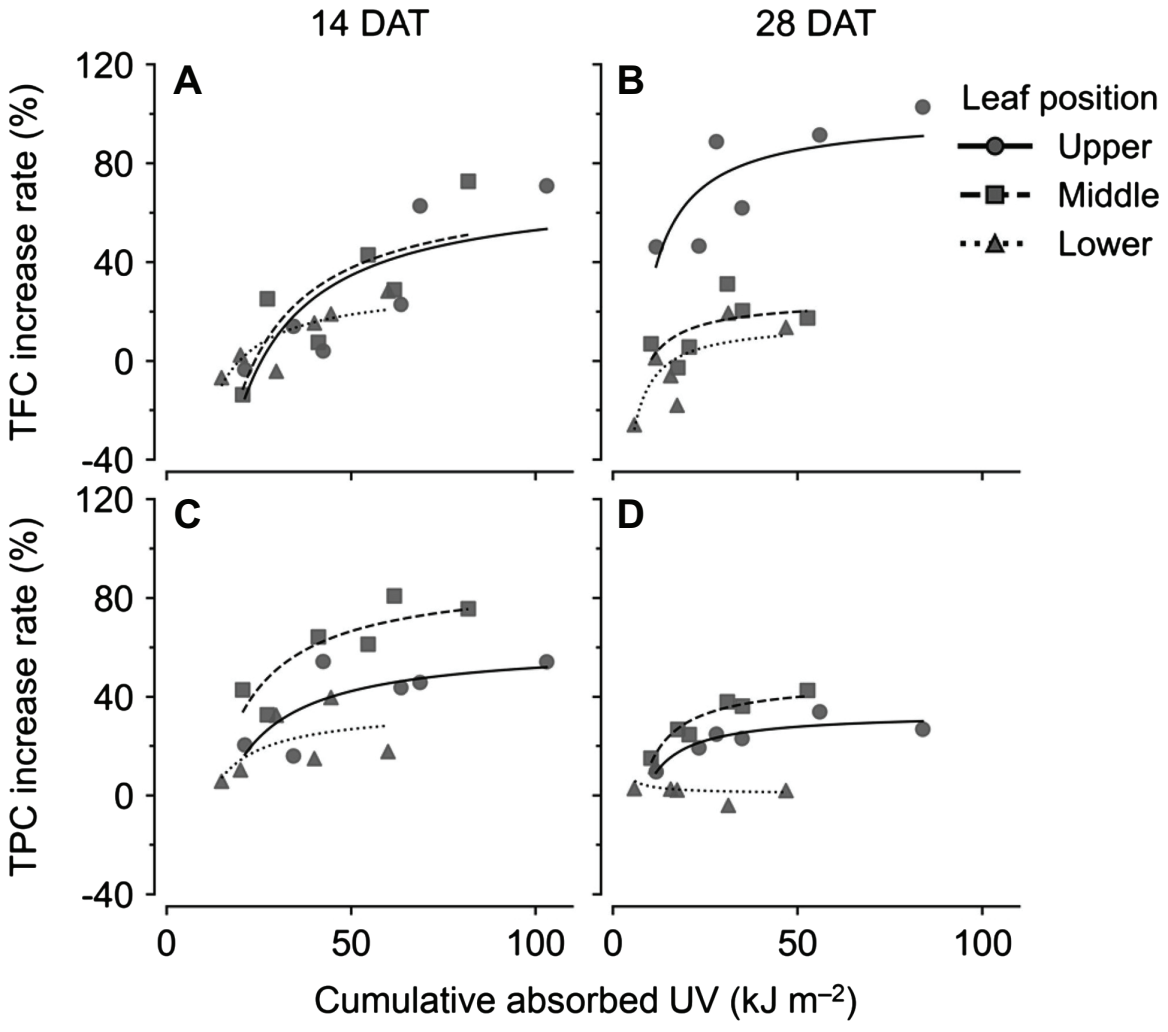
Nonlinear regression for increase rates of compound concentrations compared to the control with cumulative absorbed UV for 3 days of kales according to leaf positions and growth stages; total flavonoid compounds (TFC; **A,B**) and total phenolic compounds (TPC; **C,D**) at 14 **(A,C)** and 28 **(B,D)** days after transplanting (DATs). The cumulative absorbed UV means the integration of UV irradiation absorbed by each leaf for 3 days. Refer to Eq. 2 for the nonlinear regression equation, and Table 1 for R^2^ of the nonlinear regression.

Changes in TFC and TPC levels relative to cumulative absorbed UV amounts were regressed according to leaf positions and growth stages using rectangular hyperbolic equations (Figure 7; Eq. 2). The *R*^2^ values of these nonlinear regressions are shown in Table 1. The patterns of increase rates, i.e., the cumulative UV energy yields, were dependent on the type of bioactive compound. UV yields for TFC were highest in the upper leaves at 28 DAT (Figure 7B), but those among leaf positions did not differ noticeably at 14 DAT (Figure 7A). On the other hand, UV yields for TPC were highest in the middle leaves at both growth stages and decreased for all leaf positions at 28 DAT compared to those at 14 DAT (Figures 7C,D).

## Discussion

### Effect UV-B Radiation on Plant Growth

In most previous studies, growth inhibition in UV-B-acclimated plants was observed for numerous species (Hofmann et al., 2001; Jansen, 2002; Hectors et al., 2007). As growth progressed, all growth characteristics under UV-B exposure were slightly lower than those of the control (Figure 2). In addition, *F_v_*/*F_m_* values for all treatments remained at 0.82–83, which indicated normal growth conditions (Baker, 2008). In this study, the UV-B_BE_ doses were 2.1 or 4.2 kJ m^−2^ d^−1^ in UV_6 h_ and UV_12 h_ treatment, and the daily light integral in the PAR range was 11.5 mol m^−2^ d^−1^. These unrealistic ratios of UV-B_BE_ and PAR for a day are far from those under sunlight (Bornman et al., 2019). The photon ratio of 1.3% between the UV-B (not UV-B_BE_) and PAR also differed from sunlight, which is less than 0.2% (Robson et al., 2019). Despite of the higher UV-B/PAR ratio, the short-term UV-B duration was not sufficient to affect the growth and photosystem II activity. These results were consistent with the previous results, which did not have a negative effect on the growth of kale exposed to UV-B radiation for 2–3 days before harvest (Yoon et al., 2020, 2021).

### UV-B Radiation Interception With Growth Progress

Several studies have determined the spatial distributions of light interception in plant canopies by use of a mathematical or functional structural plant model in soybean, maize, and tomato (Wells et al., 1993; Stewart et al., 2003; Sarlikioti et al., 2011). Similar to the previous results, UV radiation interception increased with leaf height and leaf position for both growth stages (Figures 3, 4). However, average light interception decreased with growth progress for all leaf positions (Figure 4B). In contrast, the previous reports for taller crops showed higher light interception than for shorter crops with growth progress (Xue et al., 2015; Cabrera-Bosquet et al., 2016). Unlike natural light, the narrow radiation ranges of artificial light sources, such as LEDs in controlled environments, affects the irradiation area depending on lighting distance and planting conditions. Calculation based on the scanned plant models, the distance of the upper, middle, and lower leaves from the light source was 29, 30, 33 cm at 14 DAT and 24, 27, 30 cm at 28 DAT, respectively (data not shown). In the center of an empty growth chambers, the light intensities of LED modules increased as the lighting distance increased from 15 to 45 cm (Hitz et al., 2019). Under the LED arranged vertically above lettuce plants, the total light interception also increased as the lighting distance increased from 20 to 35 cm (Kim et al., 2020b). Therefore, although the upper leaves at 28 DAT were irradiated closer to the UV LEDs compared to those at 14 DAT, the average light interception would have been relatively low due to less overlap of radiation. As growth progresses, higher leaf density, which called leaf area index, causes shading at the single or canopy level and reduces UV-B radiation interception along with the narrow radiation range of LEDs. In this study, planting densities used in the simulation and actual cultivation were 24 and 12 plants per one bed at 14 and 28 DAT, respectively, which correspond to 22.2 and 11.1 plants per m^2^ (Supplementary Figure S1). Due to the growth progress, the leaf area indexes were 0.40 and 1.60 calculated as total leaf area per one bed area (m^2^/m^2^). Similarly, Kang et al. (2019) found in paprika that the estimated light interception rapidly decreased with a relative increase of surrounding plants. Due to the higher leaf area index, leaf angles in the middle leaves were twisted close to 90° or overlapped with each other and thus prevented irradiation. Similar to *Arabidopsis*, which has spiral phyllotaxis leaves at intervals of 137.5°, kale’s overlapping leaves within individual plants affect light interception patterns (Strauss et al., 2020). Therefore, these results showed that the growth progression, along with self-shading or neighboring plants, alters the positional distributions of light interception under UV LEDs.

### Phenolic Compound With UV-B Radiation Interception

UV-B has been used to irradiate various crops to enhance bioactive compound contents in controlled environments (Czégény et al., 2016; Yavaş et al., 2020). In this study, the longer daily time and duration of UV-B exposure, i.e., greater cumulative UV-B dose, induced higher TFC and TPC levels (Figure 5). Similarly, a UV-B dose-dependent response of biosynthesized secondary metabolites was found in St. John’s wort and sweet basil with continuous or repeated UV-B exposure (Brechner et al., 2011; Mosadegh et al., 2018). However, the UV-dose dependence of TFC and TPC at 28 DAT appeared only in the upper leaves, which were exposed to higher UV-B intensities (Figures 4, 5). As growth progressed, similar to the vertical distribution of light interception, the differences in UV-B-induced TFC and TPC among leaf positions became highly distinct (Figure 5). This result suggested that the patterns of bioactive compound accumulation were affected by UV radiation interception patterns according to plant structure. A previous study indicated that flavonol profiles were highly associated with characteristics that were related to canopy structure and light interception (Martínez-Lüscher et al., 2019). One of the main flavonoids, catechins, in an albino tea cultivar, significantly decreased with plant shading, which was associated with DNA methylation involved in the flavonoid biosynthesis pathway (Xu et al., 2020). In this study, RSA levels in the upper leaves were also highest for both growth stages (Figures 5E,F). Antioxidant capacity is an indicator that can indirectly show a plant’s physiological ability of its leaves (Blum-Silva et al., 2015). Phenolic acids and flavonoids containing catechol structures have been highly correlated with their radical scavenging capacity (Ayaz et al., 2008; Zietz et al., 2010; Fiol et al., 2012).

### UV-B Stress Susceptibility According to Leaf Developmental Age

Developmental age of leaves, as well as UV-B energy, determines antioxidant capacity and flavonoid accumulation, and thereby cause within-individual heterogeneity of UV-B response (Csepregi et al., 2017; Yoon et al., 2021). Heinze et al. (2018) reported that phenolic and hydroxycinnamic acids accumulated to higher levels in plants with mature leaves than in plants with younger leaves, which indicated that they can also be applied to mature and young leaves in individual plants. In addition, the growth progress caused large variations in leaf age within plants. In this study, TFC and TPC levels in lower (older) leaves at 28 DAT showed the lowest values among treatments (Figures 5C,D). RSA levels in lower leaves at 28 DAT were significantly lower than those at 14 DAT (Figures 5E,F). Even for the same position, the leaf ages of the lower leaves were at least 2 weeks at 14 DAT and were at least 4 weeks at 28 DAT. Similarly, younger leaves also exhibited higher antioxidant capacities than older leaves in grapevines (Majer and Hideg, 2012). The linear correlations between TPC and TFC levels and cumulative UV absorbed for 3 days were clearly distinguished by leaf position and age (Figure 6). The gradient of the regression line, i.e., the increase rate of TPC and TFC against UV energy, was interpreted as the age-dependent UV-B stress susceptibility (Figure 7). As growth progressed, the TFC increase rate in the upper leaves increased noticeably (Figure 7B). In other words, young leaves had the highest UV-B susceptibility for TFC, which was consistent with the previous findings of general stress susceptibility with leaf age (Sperdouli and Moustakas, 2014; Moustaka et al., 2015; Kanojia et al., 2020). As growth progressed, the TFC and TPC increase rates increased or decreased for all leaf positions, but the actual concentrations were similar between 14 and 28 DAT (Figures 5, 7). These results were caused by both higher UV-B light interceptions at 14 DAT and higher concentrations with growth progress in the control without UV-B exposure. This tendency is consistent with the results of a previous study, which showed the increases in TFC, TPC, and total glucosinolate contents in kale without UV-B exposure in plant factories (Yoon et al., 2019).

### Potential of UV Energy Yield for Phenolic Profiles in 3D Plant Structure

In general, the radiation use efficiency is obtained as the slope of the regression line for biomass accumulated with PAR light intercepted by a plant (Chakwizira et al., 2014). Accordingly, the slope of the regression line of the accumulated TPC or TFC content with absorbed UV energy can be regarded as UV energy yield (Figures 6, 7). Canopy distributions of nitrogen (N) and phosphorus, whose levels remained constant or gradually decreased in aging leaves, were inferred to be related to UV energy yields for TFC and TPC (Coleman, 1986; Chakwizira et al., 2011). Radiation use efficiency was determined not only by canopy N distributions but also by leaf N allocations in the thylakoid light-harvesting proteins of *Brassica* crops (Fletcher et al., 2013; Liu et al., 2020). In this study, the cumulative UV energy yields for TFC or TPC accumulation were determined by plant ages as well as by leaf ages (Figure 7). Although the actual leaf ages of the upper leaves were the same at approximately 1–2 weeks from their appearance between 14 and 28 DATs, the TFC increase rate was markedly higher at 28 DAT (Figures 7A,B). In contrast, the UV-B increase rate for TPC was highest in the middle leaves (Figures 7C,D). The result was consistent with a previous study, which showed that the TPC increase rate in the middle leaf was the highest because younger leaves had higher TPC even without UV-B exposure (Yoon et al., 2021). Similarly, UV-B-induced metabolite accumulations depended on chemical structure as well as chalcone synthase activity (Neugart et al., 2012; Ghasemzadeh et al., 2016). Despite the predominance of UV energy yield in the middle leaves at 28 DAT (Figure 7D), actual TPC concentrations were significantly higher in the upper leaves (Figure 5D). If the amount of UV radiation interception in the middle leaves was not intercepted by the upper leaves at 28 DAT, TPC concentrations in the middle leaves could be higher than those in the upper leaves. Therefore, the UV-B-induced bioactive compound contents in the plant structure can be estimated when the age-dependent cumulative UV energy yields and distributions of UV-B radiation interception are considered simultaneously.

### Application to Bioactive Compound Production in Plant Factories

Several studies about modeling the light environment perceived by the plant organs using various techniques, such as 3D plant model and Monte Carlo ray-tracing, have been focused on light interception in the PAR range (Vos et al., 2010). Recently, Kim et al. (2020a) demonstrated the structural accuracy of 3D-scanned parametric model and their application for estimating photosynthetic rates as well as light interception of sweet pepper. Kim et al. (2020b) applied the same technique to lettuce canopy in plant factories with electrical lighting, which allowed the interpretation and evaluation of light use efficiency according to planting density and lighting condition. In the same way, our previous study applied the radiation interception analysis to the UV-B range and confirmed that the UV-B radiation interception of a whole plant determines intraindividual distribution of phenolic contents in kales along with leaf age (Yoon et al., 2021). In this study, we suggested the concept of UV energy yield for phenolic contents in short-term UV-B exposure and showed its applicability to other crops with self- or neighboring shading at various plant stages in controlled environments. In a further step, the UV-induced accumulation of bioactive compounds can be estimated for various plants and lighting condition, such as wavelengths, shape, arrangement, and distance. For example, the UV sensitivity of TPC was unexpectedly highest in the middle leaves at both growth stages (Figure 7). Considering the largest proportion of the middle leaves in plants, further studies are possible on the lateral UV-B irradiation method that focuses on the middle leaves.

From a commercial point of view, maximizing production efficiency against input costs is more important than the output itself. The annual production of TPC and TFC in plant factories could be estimated using the content per plant at various growth stages without neighboring shading, which could determine the optimal harvest time (Yoon et al., 2019). Chowdhury et al. (2021) reported that health-promoting components, specifically glucosinolates and anthocyanin, in kale grown in a plant factory can be estimated through spectral reflectance. Similar to these studies, the annual production of UV-B-induced bioactive compounds can be estimated through UV-B radiation interception analysis. Ultimately, the application of 3D plant model and radiation interception analysis will enable us to evaluate the UV illumination strategies to maximize production vs. UV energy or electrical input in commercially controlled environments.

## Conclusion

The positional distributions of UV-B radiation interception and bioactive compound contents in kale leaves were quantitatively analyzed with 3D-scanned plant models and 3D light analysis. Concentrations of total flavonoid and phenolic compounds showed their highest values in the upper leaves for both growth stages. As growth progressed, variations in absorbed UV-B, as well as UV susceptibility, at each leaf position became evident. This study confirmed that biosynthesis of bioactive compounds in plant structures was determined by UV-B radiation interception and cumulative UV-B energy yield based on leaf position, leaf age, and plant growth stage. This attempt to quantitatively analyze the relationships between secondary metabolites and UV-B light interception can be applied to model bioactive compound production in plant factories.

## Data Availability Statement

The raw data supporting the conclusions of this article will be made available by the authors, without undue reservation.

## Author Contributions

HY, HK, and JS designed the research and prepared the manuscript. HY and HK performed the experiments. HY, HK, and JK analyzed the data. HY, JK, and JS revised and edited the manuscript. All authors contributed to the article and approved the submitted version.

### Conflict of Interest

The authors declare that the research was conducted in the absence of any commercial or financial relationships that could be construed as a potential conflict of interest.
