# Supplementary material for: Quantitative Analysis of UV-B Radiation Interception and Bioactive Compound Contents in Kale by Leaf Position According to Growth Progress
Source: Front Plant Sci. 2021 Jul 8;12:667456. doi: 10.3389/fpls.2021.667456 (PMC8297650; doi:10.3389/fpls.2021.667456)
Supplement: Supplementary file 1 [file Data_Sheet_1.PDF]

Supplementary Material

1 Supplementary Figures

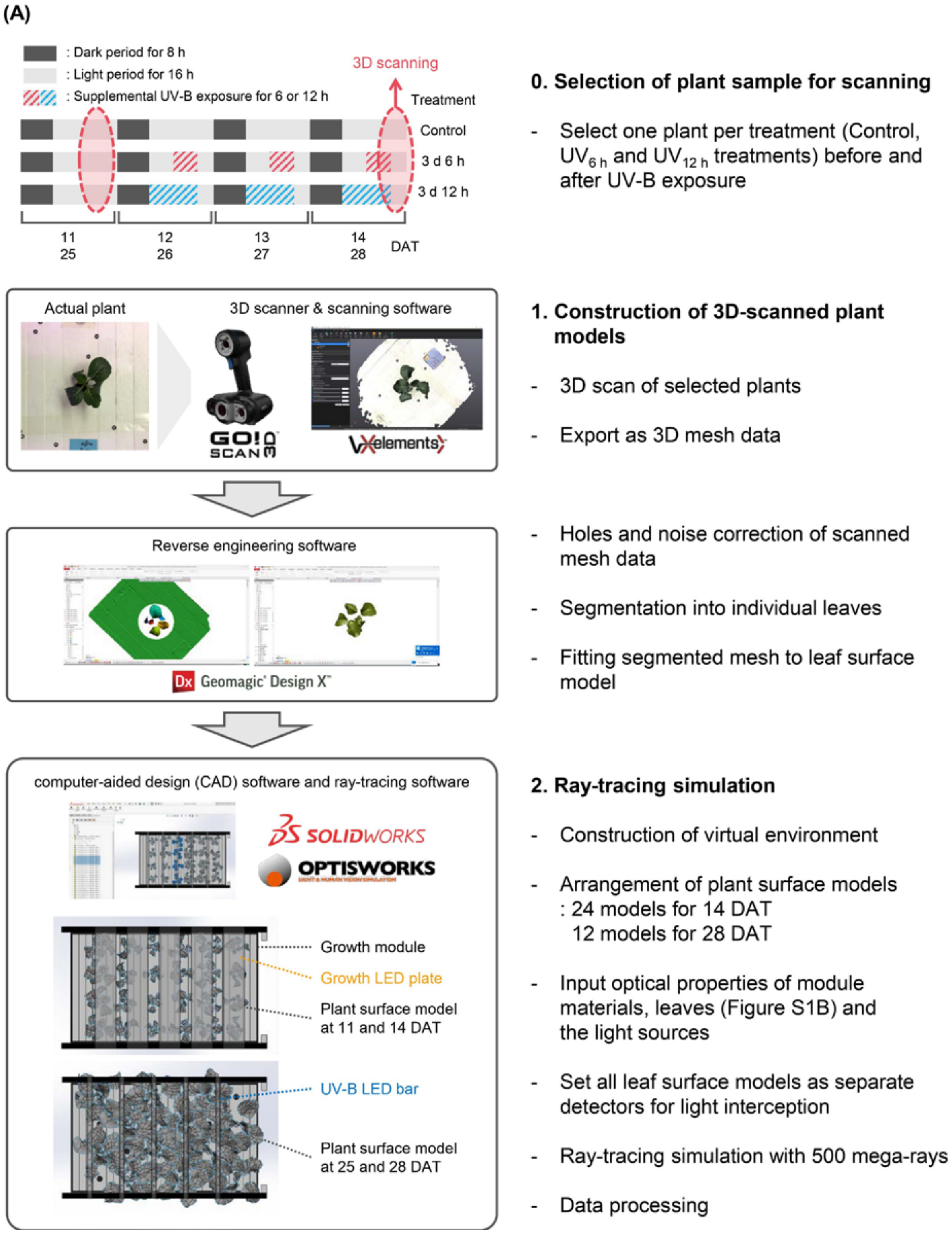

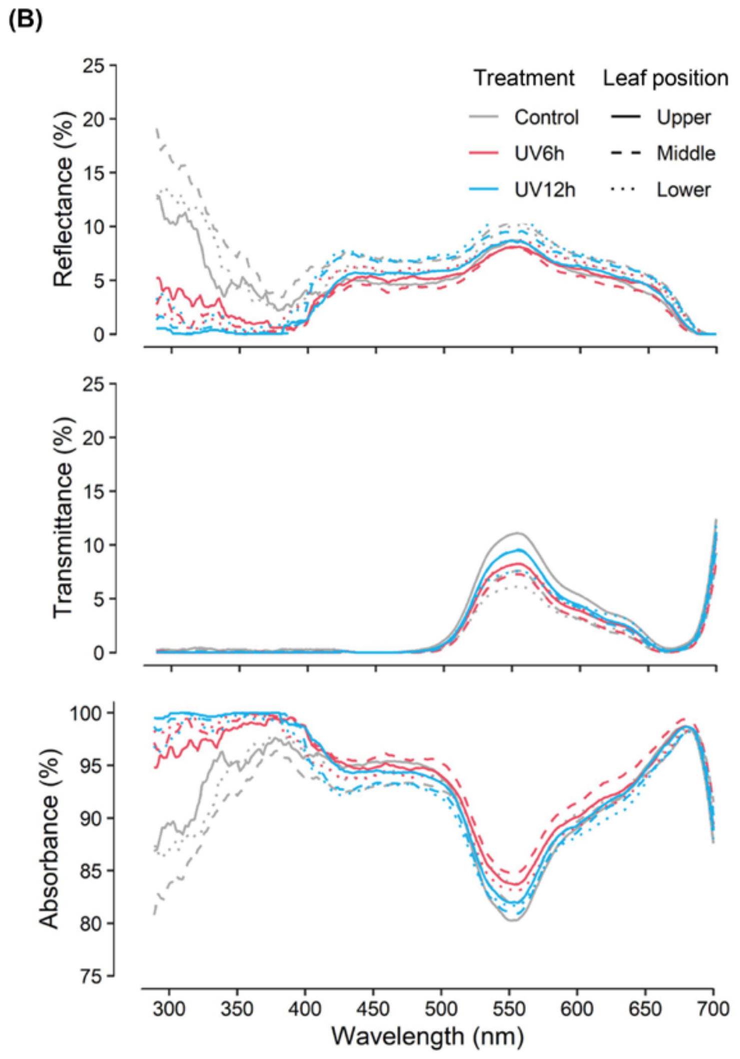

Figure S1. The detailed procedure and condition from 3D scan to simulation in 2.6 section **(A)** and optical properties of kale leaves according to UV-B treatment and leaf position **(B)**.
